# Material analysis on semi-permanent makeup needles

**DOI:** 10.1186/s42649-024-00103-1

**Published:** 2024-11-16

**Authors:** Hyun Sook Jin, Seung Hyun Oh, Byung Soo Chang

**Affiliations:** 1 Department of Cosmetology, Hanseo University, 46 Hanseo 1-ro, Haemi- myeon, Seosan, Chungnam 31962 South Korea; 2 Department of Fashion Management, Fashion Institute of Technology, SUNY Korea, Incheon, 21985 South Korea; 3 Department of Cosmeology, Hanseo University, 46 Hanseo 1-ro, Haemi- myeon, Seosan, Chungnam, 31962 South Korea

**Keywords:** Cosmetics, EDS, Make up, Microblading, Micro-pigmentation, SEM, Semi-permanent makeup, Tattooing, Tattoo needle

## Abstract

The cosmetic-tattoo industry is evolving every year and the microstructures of the equipment have the great potential for semi-permanent makeup applications. Present paper explores the materials and microparticles of semi-permanent makeup tattoo needles. The surface of the five-round-shader tattoo needle used in semi-permanent makeup process was examined by scanning electron microscopy, and its elemental composition was analyzed by energy-dispersive X-ray spectroscopy. The comparison of five-round-shader needles have undergone thorough observation: original five-round-shader needle and distorted five-round-shader needle. The diameter of the sharp and rounded needle tip was measured at approximately 6.80 μm, while the deformed needle tip was approximately 16 μm thick, about 2.5 times thicker than the rounded needle tip. Many rosette-shaped lead (Pb) particles and irregular clusters adhere to the welded areas and closely adjacent needle shaft surfaces. The lead particles have a diameter ranging from 4 μm to 5 μm and exhibit a grid-like structure with a consistent thickness of plate-like shape. The distorted structure of Pb in rosette-shaped formations is shown to have originated from the grinding and polishing processes during needle manufacturing. To produce sterilized tattoo needles, high-quality tattoo needle inspection processes are necessary to remove any unhygienic substances adhering to the needle surface.

## Introduction

Humanity has been practicing tattoos for thousands of years, dating back to the Stone Age. Tattoos have served as a form of self-expression, allowing individuals to showcase their personality, beliefs, interests, or experiences displayed on their bodies: indicating empowerment, cultural or religious significance, and symbol of punishment. In addition, tattoo was practiced for aesthetics purposes to cover skin wounds or scars, enhancing physical beauty (Dorfer et al. 1999).

The symbolism behind tattoo has evolved over time for its social norms and cultural attitude. Once regarded as symbols of rebellion or nonconformity, tattoos, much like piercings, have evolved into expressions imbued with deeply personal significance, particularly among the younger generations in Europe and North America (Nishioka and Gyorkos 2001).

The terminology of tattoo originated from the Tahitian language and is currently used in various terms such as semi-permanent makeup, cosmetic tattooing, micropigmentation, contour makeup, permanent makeup, and scalp micropigmentation. This encompassed a range of terms depending on the scope of tattooing procedures, skin treatment depth, and purposes (AlQuorain et al. 2017).

Recently, semi-permanent makeup has become prevalent among women for its convenience - eyeliner, eyebrows, lip liner, full lip pigment, cheek blush, and beauty marks - to enhance facial features without the need for daily makeup application (Singh and Karki 2010; AlQuorain et al. 2017).

In Korea, semi-permanent makeup has become popular for enhancing the shape of eyelids, enlarging or correcting the position of eyebrows, and improving the contour of distorted lips (Jin and Chang 2019; Jin and Chang 2022a).

Semi-permanent makeup, also known as cosmetic tattooing, is a complexion treatment to enhance eyes, brows and lips to add definition to the face (Fulton Jr et al. 2000). This technique involves injecting pigment through a fine vibrating needle into the most superficial layers of the skin. The color pigment only penetrates 0.2 mm deep and unlike a tattoo it fades with time. The benefits of semi-permanent treatment Injecting pigments into the eyebrows, eyeliner, and lips add color to the lips and help balance it out, enhancing the overall aesthetics. Therefore, semi-permanent lip treatment adds color and adjusts irregular shape for a more natural and healthier look, while eyebrow treatment provides a more defined look after the procedure.

Lip tattooing is a simple and long-lasting technique compared to injectable dermal filler into the lips to restore color and plumpness just like French lips. Lip tattooing procedure involves inserting pigment and/or tattoo ink into your skin with small sterile tattooing needles based on the customer’s preference of color and shape. Red pigment is used for the treatment for a glossy full lip (Fulton Jr et al. 2000).

Semi-permanent eyebrows treatment, filling in empty areas with pigment into the top layer of skin to create the illusion of eyebrow hair, dramatically enhances the beauty of a face by complementing the eyes and creating a youthful appearance with a more long-lasting natural eyebrow effect (Oumeish 2001).

The benefit of semi-permanent makeup is less time a simple, yet satisfactory results for clients require extensive training and experience over time. Before the procedure, it is crucial to select the appropriate design (shape and color) for the client, and aftercare is essential to prevent infections and complications and to maintain the tattooed area as desired (Wenzel et al. 2016; Jin and Chang 2022b). Furthermore, the creation of tailored eyebrow requires a skilled technician who understands the client’s natural hair pattern and uses a fine blade to manually deposit pigment into the skin.

In semi-permanent makeup procedures, the needle is a tool that penetrates through the skin barrier and into the dermal papilla layer. Pigments, which can produce various colors, are sufficiently embedded in the tip and some parts of the body of the needle so that when the skin is torn or torn, they can move down to the dermal papilla layer.

Semi-permanent makeup procedures mostly involve delicate work targeting facial skin. Practitioners must maintain precision using manual techniques, either working directly with their hands or indirectly using tools. The material of the needle is steel, which has been primarily researched for its use in piercing the human skin (Xie et al. 2014).

The procedure of semi-permanent makeup includes manual techniques where the needle is inserted into a tattoo pen, machine techniques where it’s attached to equipment, embroidery techniques where it’s applied similar to stitching, and combination techniques which involve a mix of these methods (Jin et al. 2023).

The inherent risk of infection is always present during the process of semi-permanent makeup procedures. Despite disinfecting the skin of the treated area and taking appropriate preventive measures, skin deposition of metallic substances attached to the needle or microbial contamination can occur post-procedure (Jin and Chang 2022b; Rello et al. 2022).

The risk of infection varies depending on several determining factors such as the condition of the skin undergoing semi-permanent makeup, proper disinfection of equipment (Kennedy et al. 2012; Jin and Chang 2019; Kang and Chang 2023), contamination of ink products, skin disinfection, and appropriate post-care management (Høgsberg et al. 2013; Jin and Chang 2020).

There have been reports on the needle tips after semi-permanent treatments (Schreiver et al. 2019; Jin and Chang 2022b). Schreiver et al. (2019) reported wear on the needles before and after tattooing procedures on pig skin, while Jin and Chang (2022b) observed the flattened shape of 12 needles using a scanning electron microscope, confirming their defective condition. The five-round-shader is widely used for its natural shading purposes. Jin and Chang (2022b) reported that needles used in semi-permanent makeup procedures often have bent or flattened tips, resembling a mushroom shape. They warned that during the procedure, tiny metal fragments may detach from the needle tip and become embedded in the skin.

Korea’s beauty industry has become one of the country’s new growth engines. With the rapid development of innovative materials and technology, the sophisticated technique of semi-permanent makeup procedures has achieved one of the top 10 beauty markets in the world. The current Korea’s legal system considers cosmetic tattooing as a medical practice and only allows the practice be carried out by technicians with a medical license. On the contrary, the rigid regulation is only applied to the license not equipment and supplies as it is categorized as consumer goods instead of medical devices by Korean Ministry of Food and Drug Safety. In reality, there are concerns about the insufficient regulation of quality control for needles and pigment used for the procedure.

This study observed the attachment of metal materials and foreign substances on the surface of needles produced and used as consumer goods in Korea using scanning electron microscopy (SEM). Additionally, it analyzed the elemental composition of these foreign substances using energy dispersive X-ray spectroscopy (EDS). The aim of this research is to analyze and bring awareness contribute to the production of high-quality needles by identifying causal factors of skin inflammation and allergic reactions that may occur after semi-permanent makeup procedures.

## Materials and methods

### Sample Processing

Five-round-shader sterilized tattoo needles, commonly used in semi-permanent makeup procedures, were purchased and utilized as experimental materials. The purchased needles were directly unpacked without any treatment before use as experimental materials.

**Fig. 1:**
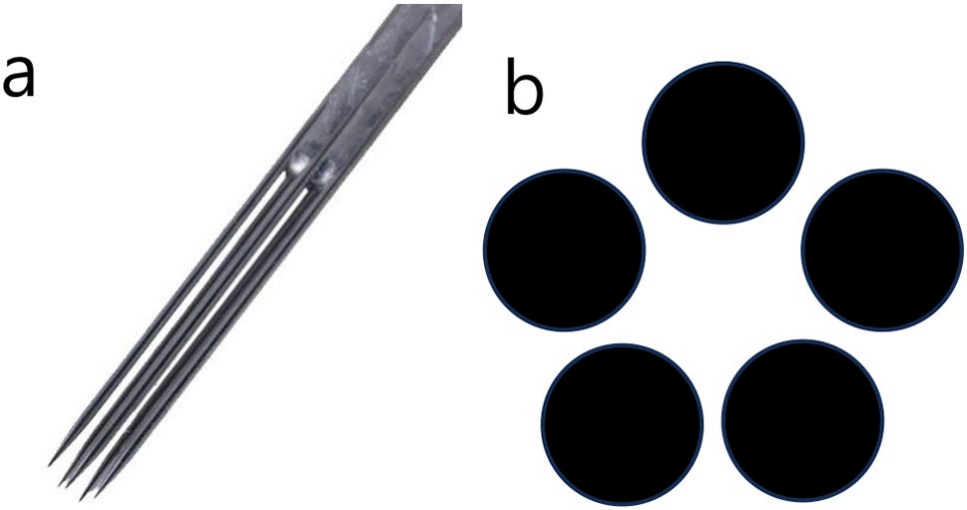
Five-round-shader tattoo needle (**a**) and the shade with its use (**b**)

### Electron microscopic analysis

To examine the microstructural morphology of the body and tip surface of the five-round-shader sterilized tattoo needle, 4 cm length of the needle was cut into 1.5 mm sections using a sharp cutting machine. The samples were then mounted onto stubs treated with carbon tape and copper tape. Subsequently, they were platinum-coated to a thickness of 20 nm using an ion coater (IB-5 ion coater, Eiko, Japan), and observed under a scanning electron microscope (S-4700, Hitachi, Japan) at 15 kV.

### Energy-dispersive X-ray spectroscopic analysis

The stub with appropriately sized sections of the five-round-shader tattoo needle attached was mounted onto the ion coater (IB-5 ion coater, Eiko, Japan) and platinum-coated to a thickness of 20 nm. Subsequently, analysis was conducted using energy dispersive X-ray spectroscopy (INCA, Oxford Inc., Great Britain). The acceleration voltage used for this analysis was 15 kV.

## Results

**Fig. 2:**
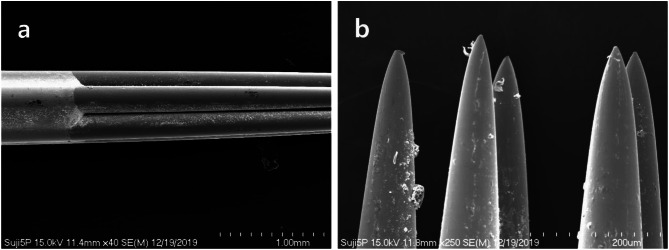
Low magnification scanning electron micrograph of five-round-shader tattoo needle body (**a**) and tip(**b**)

Five-round-shader sterilized tattoo needles used in semi-permanent makeup procedures were observed under a scanning electron microscope. In the low-magnification observation, numerous foreign substances were found attached to the surface of the needles. Additionally, many foreign substances were observed attached to the welded areas and body surfaces of the needles. The welded areas of the needles where the five needles were tightly joined were observed to be clean without any rough surfaces (Fig. 2a).

Under 100x magnification of the scanning electron microscope, foreign substances at the tip of the needle appeared in clumps or powdery particle forms, and deformation of the needle tip shape was also observed. However, the surface of the metal column itself was observed to be smooth, without any scratches or grooves (Fig. 2b).

**Fig. 3:**
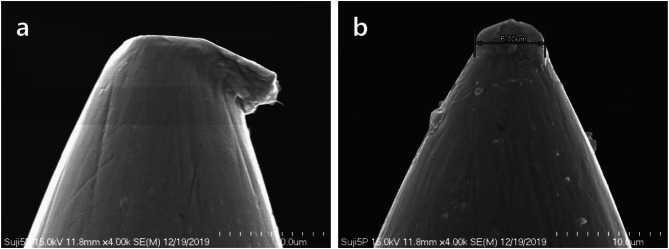
Scanning electron micrograph of (**a**) a distorted (**b**) a poorly made five-round-shader tattoo needle tip

Under high-magnification scanning electron microscope observation, the tips of the five-round-shader tattoo needles exhibited deformed shapes, including bent (Fig. 3a) and rounded (Fig. 3b) forms. The diameter of the distorted needle tip in Fig. 3a was approximately 16 μm, while the diameter of the rounded needle tip in Fig. 3b was measured at 6.80 μm.

**Fig. 4:**
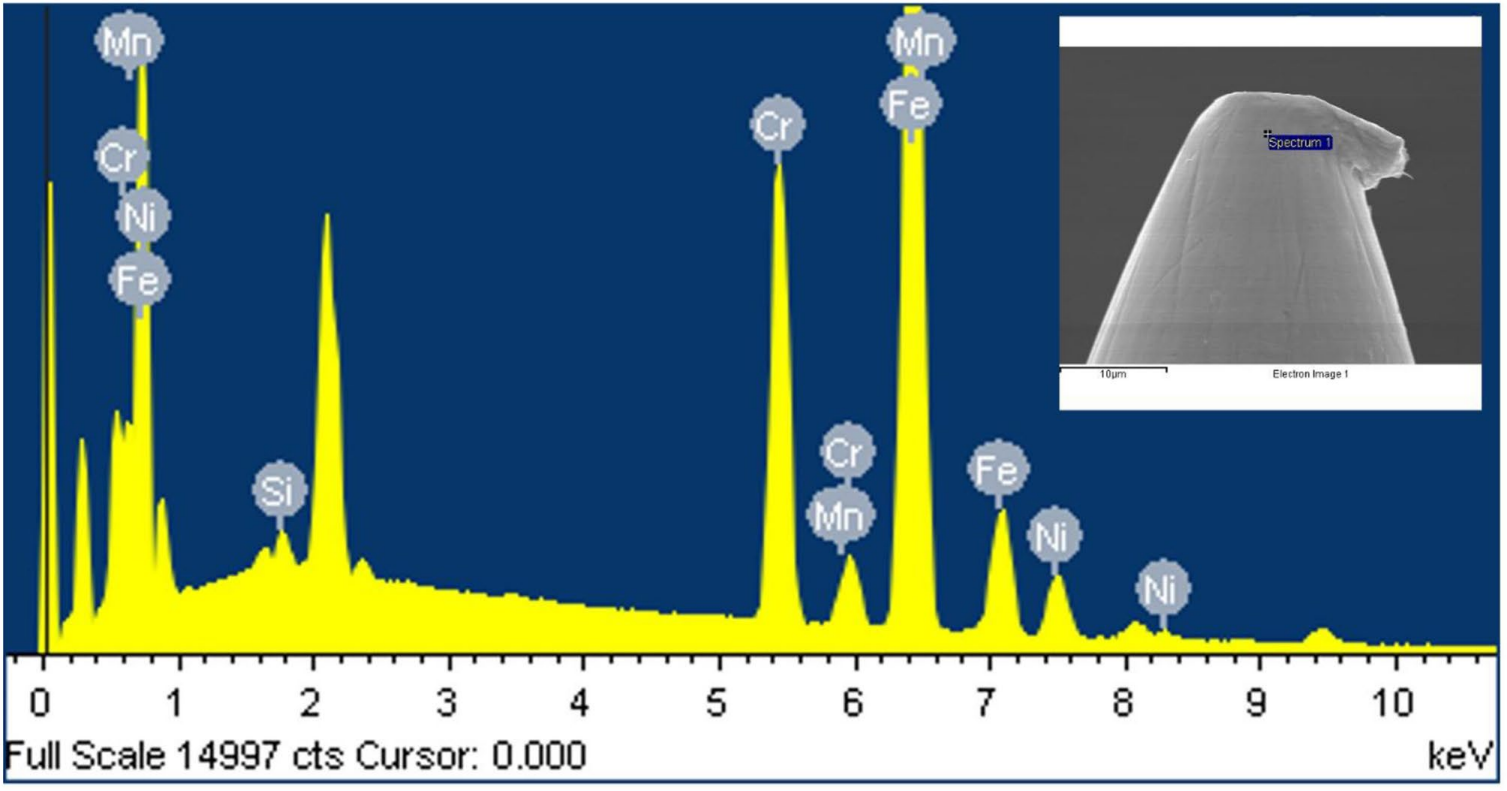
Energy-dispersive X-ray spectroscopy (EDS) spectrum of the five-round-shader tattoo needle tip sample. EDS reveals Fe, Cr, Ni, Mn, Si elements. Inset: Content of the constituent elements of needle tip

To determine the elemental composition of the five-round-shader tattoo needle, the surface of the needle was analyzed using energy dispersive X-ray spectroscopy (EDX). The results revealed the presence of iron (Fe), chromium (Cr), nickel (Ni), manganese (Mn), and silicon (Si) elements (Fig. 4). The elemental composition was found to be 70.75% Fe, 19.79% Cr, 7.43% Ni, 1.12% Mn, and 0.91% Si (Table 1).

**Table 1:** Elemental composition of needle material by Energy-dispersive X-ray spectroscopic analysis

Element	Weight %	Atomic %
Si K	0.47	0.91
Cr K	18.69	19.79
Mn K	1.11	1.12
Fe K	71.80	70.75
Ni M	7.93	7.43
Total	100.00	100.00

**Fig. 5:**
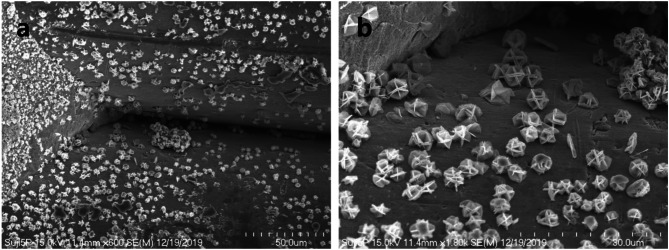
Scanning electron micrograph of many flower-shaped particles of five-round-shader tattoo needle. (**a**) Amorphous substances seen between the flower-shaped particles. (**b**) Magnification scanning electron micrograph of Fig. (4a) shown as many flower-shaped particles

Numerous particle-like substances and irregular clumps are attached to the welded areas and surfaces of adjacent needle columns of the five-round-shader tattoo needle five-round-shader tattoo needle (Fig. 5a). The shapes of these particles were not accurately identified under low-magnification observation, but under high magnification, the particle-like substances were observed to have formed into regular shapes resembling rose petal-like structures (rosette form) (Fig. 5b).

**Fig. 6:**
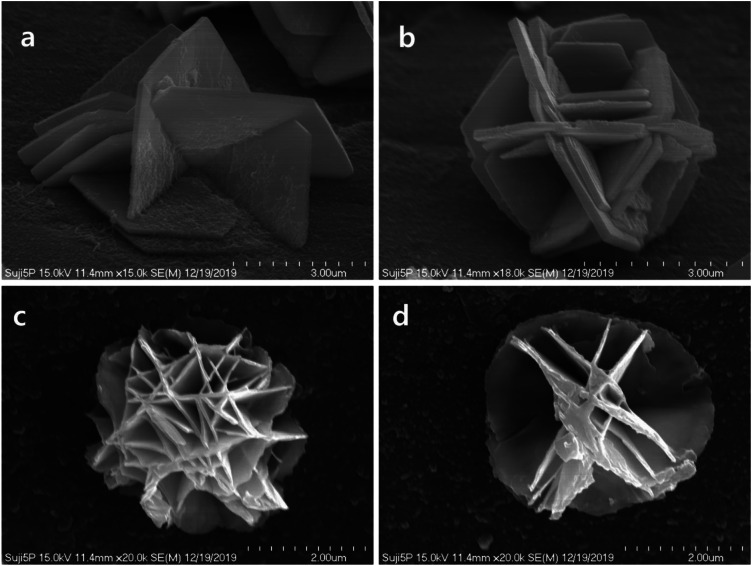
High magnification scanning electron micrograph of a lattice structure in the flower shaped particle. (**a**)-(**d**): flower-shaped lead particles

Under high-magnification scanning electron microscopy, the diameter of the petal-shaped particles was observed to be between 4 μm and 5 μm, exhibiting a grid-like structure. These grid structures were present as structures of plates with uniform thickness (Fig. 6).

**Fig. 7:**
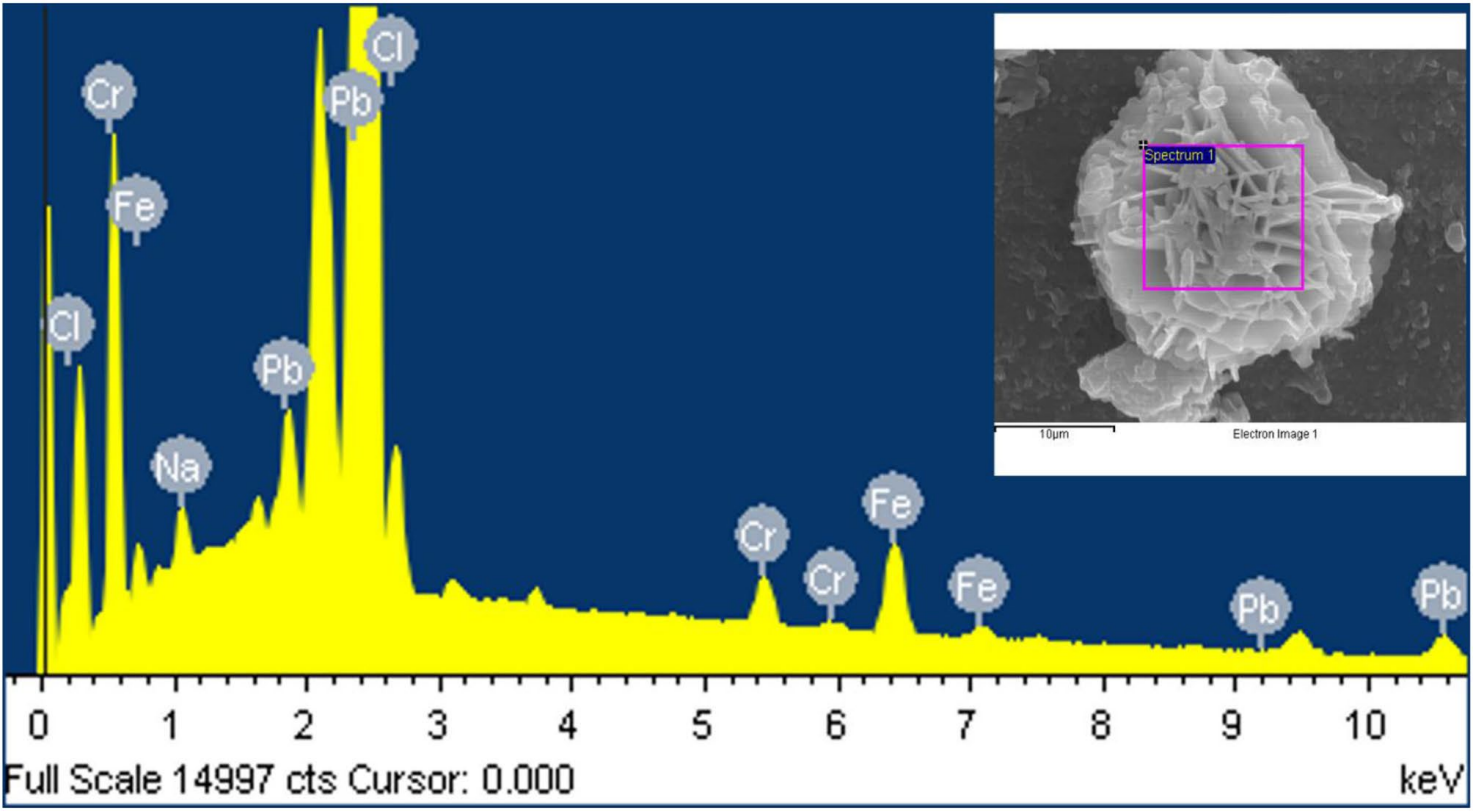
EDS spectrum of the flower shaped particle of five-round-shader tattoo needle surface sample. EDS reveals Pb, Fe, Cr, Cl, Na elements. Inset: Content of the constituent elements of flower shaped particle of needle surface

Energy dispersive X-ray spectroscopy (EDX) analysis conducted to determine the elemental composition of the petal-shaped particles observed on the surface of the five-round-shader tattoo needle revealed the presence of lead (Pb), iron (Fe), chromium (Cr), chlorine (Cl), and sodium (Na) elements (Fig. 7).

Lead (Pb) was detected in proportions of 61.55%, iron (Fe) at 18.65%, chromium (Cr) at 7.09%, chlorine (Cl) at 6.45%, and sodium (Na) at 6.27%, as shown in Table 2. Based on these elemental composition ratios, the particle-like substances present on the welded areas and body surface of the five-round-shader needle were confirmed to be lead particles.

Table 1. Energy-dispersive X-ray spectroscopy analysis of the elemental composition of content of the constituent elements of needle tip.

**Table 2:** Elemental composition of needle material by Energy-dispersive X-ray spectroscopic analysis

Element	Weight %	Atomic %
Na K	0.99	6.27
Cl K	1.57	6.45
Cr K	2.54	7.09
Fe K	7.17	18.65
Pb M	87.73	61.54
Total	100.00	100.00

## Discussion

The needles used in semi-permanent makeup are crucial tools used to penetrate the skin during the treatment. It is commonly believed by the general public that needles are sharp and have rounded tips. Needles with abnormal or deformed shapes may cause pain at the application site during tattooing procedures and can potentially lead to skin disorders if metallic particles are shed from the needle.

In this study, some of the five-round-shader tattoo needles were observed to have a slightly rounded shape, while others exhibited deformation at the tip. The needle tip with a rounded shape had a diameter of 6.80 μm, while the deformed needle tip measured approximately 16 μm, making it about 2.5 times thicker than the rounded needle tip. Proceeding with tattooing procedures using deformed needles can lead to the deposition of metal fragments deep into the dermis, causing skin inflammation due to their residue (Rubianes and Sanchez 1993; Kazandjieva and Tsankov 2007; Forte et al. 2009).

Jin and Chang (2022b) reported that the deformed structure of the needle tip appeared to have been formed during the manufacturing process of the needles. They suggested that if careless handling occurred after the production of the needles but before their use, resulting in the application of external force to the needle tip, it could lead to deformation in the impacted area of the needle tip.

The deformed characteristics of the needle tips used in this study exhibit irregular shapes such as blunt ends, protruding fragments, and other irregularities. Xie et al. (2014) reported common characteristics of acupuncture needles, including scratches, blunt ends, malformed ends, and lump formations. Additionally, Hayhoe et al. (2002) noted that needle tips often exhibited a deformed shape, with the end deviating from the center in a lumped and malformed manner.

In this study, the needle tips used for semi-permanent makeup exhibited more severe deformities compared to acupuncture needles reported by Hayhoe et al. (2002) and Xie et al. (2014).

Analysis of the elemental composition of the five-round-shader tattoo needle revealed the presence of iron (Fe), chromium (Cr), nickel (Ni), manganese (Mn), and silicon (Si) elements. These elemental components indicate that the needles are made of stainless steel. Typically, stainless steel is an alloy of steel with a chromium (Cr) content of over 11% by mass (Espy 1973; Lo et al. 2009).

The use of chromium oxide (Cr2O3) as a component in stainless steel alloys can indeed help prevent corrosion of the steel surface, although it may not be entirely effective under all conditions. However, chromium (Cr) alloyed steel is known to exhibit significantly less rusting and corrosion compared to ordinary steel (Lo et al. 2009).

In this study, the needles contained a significant amount of chromium (Cr) following iron (Fe), and they did not include other heavy metal elements.

On the surface of the welded areas of the five-round-shader tattoo needle, numerous particle-like substances were observed to be attached, and these substances appeared in the form of very fine powder particles.

In the Machine-R3p needle, the composition of the welded area where three needles are joined primarily consisted of tin (Sn) elements, with a very small amount of iron (Fe) detected.

Belfatto (2019) reported that welding in needles used for cosmetic tattooing typically involves the use of silver or tin, depending on the quality of the needle. In this research, the material used for welding was confirmed to be tin based on Energy Dispersive X-ray Spectroscopy (EDS) analysis. Additionally, tin (Sn) was detected as the elemental component attached to the surface of the needle body and the needle tip. These results suggest that the tin used in welding was dispersed and attached to the needle surface in the form of fine particles during the process.

The attached areas of the five-round-shader tattoo needle, connected to the body of the needle, is observed to have numerous peculiar particle-like substances and irregular clusters attached. The irregular attachments were confirmed to be tin material based on elemental composition analysis. However, under high magnification scanning electron microscopy observation, the particle-like substances were observed in the form of rosette-shaped structures. Their structure comprises plate-like structures arranged perpendicular or diagonally, forming a grid-like pattern.

The composition analysis of the rosette-shaped structures found on the five-round-shader tattoo needle was lead (Pb) as the major component. The particle-like substances present on the needle surface were confirmed to be lead particles.

The result of lead (Pb) on the needle surface and welded areas is mostly attributed to grinding and polishing processes during needle manufacturing.

These grinding and polishing processes generate electrostatic forces that attract small metal particles to the needle surface (Xie et al. 2014). To manufacture safe needles, proper cleaning processes are essential to remove foreign substances attached to the needle surface.

## Conclusion

In the present study, the five-round-shader tattoo needle shows significant attachment of foreign substances on the welded areas and body surface. Material in the importance placed on these items as the sessions progressed were monitored. Some needle tips are deformed and bent, with the diameter of the crushed needle tip apex measuring approximately 16 μm, which is 2.5 times larger than that of a normal needle tip. The five-round-shader tattoo needle is composed of iron (Fe), chromium (Cr), nickel (Ni), manganese (Mn), and silicon (Si) elements, with no other heavy metal substances detected. Many foreign substances adhere to the welded areas and adjacent needle surfaces, taking the form of lead (Pb) particles in rosette-shaped structures. The lead particles exhibit a diameter ranging from 4 μm to 5 μm and have a grid-like pattern on the surface. Future studies must accumulate more case studies in other professional fields and industries, and develop the indices for technological innovation that could be derived from material advancements, as well as the bases for evaluation methods. Moreover, the current research on lead particles will help understand the substance of the current equipment and further developments with reference to the next-generation technologies.

## Data Availability

Data and materials are available on request.

## Abbreviations

EDS: Energy-dispersive X-ray spectroscopy
SEM: Scanning electron microscope
